# Use of exercise tests in primary care: importance for referral decisions and possible bias in the decision process; a prospective observational study

**DOI:** 10.1186/s12875-014-0182-9

**Published:** 2014-11-30

**Authors:** Gunnar Nilsson, Thomas Mooe, Lars Söderström, Eva Samuelsson

**Affiliations:** Department of Public Health and Clinical Medicine, Umeå University, Umeå, Sweden; Unit of Research, Education and Development, Östersund Hospital, Östersund, Sweden

**Keywords:** Coronary disease, Exercise test, Primary care, Referral

## Abstract

**Background:**

The utility of clinical exercise tests depends on their support of treatment decisions. We sought to assess the utility of exercise tests for the selection of primary-care patients for referral to cardiologic care, and to determine whether referral decisions were biased by gender or socioeconomic status. We also evaluated referral rates and cardiovascular events in patients with positive exercise tests.

**Methods:**

We designed a prospective observational study of 438 men and 427 women from 28 Swedish primary-care clinics who were examined with exercise testing for suspected coronary disease. All participants were followed-up with respect to cardiologist referrals and cardiovascular events (hospitalisation for unstable angina, myocardial infarction, and cardiovascular death) within six months and revascularisation within 250 days. Variables associated with referral were identified by multivariable logistic regression. Socioeconomic status was determined by educational level and employment.

**Results:**

Positive/inconclusive exercise tests and exertional chest pain predicted referral in men and women. Of 865 participants, patients with positive, inconclusive, or negative exercise tests were referred to cardiologists in 67.3%, 26.1%, and 3.5% of cases, respectively. Overall, there was no significant difference in referral rates related to gender or socioeconomic level. Self-employed women were referred more frequently compared to other women (odds ratio (OR) 3.62, 95% confidence interval (CI) 1.19-10.99). Among non-manual employees, women were referred to cardiologic examination less frequently than men (OR 0.40, 95% CI 0.16-1.00; p = 0.049; ORs adjusted for age, exertional chest pain, and exercise test result). In patients with positive exercise tests, the referral rate decreased continuously with age (OR 0.48, 95% CI 0.23-0.97; adjusted for cardiovascular co-morbidity). Cardiovascular events occurred in 22.2% (4/18) of non-referred patients with positive exercise tests; 56% (10/18) of these patients were not considered for cardiologic care, with continuity problems in primary care as one possible contributing cause.

**Conclusions:**

Exercise tests are important for selecting patients for referral to cardiologic care. Interactions related to gender and socioeconomic status affected referral rates. In patients with positive exercise tests, referral rates decreased with age. An increased awareness of possible bias regarding age, gender, and socioeconomic status, which may influence medical decisions, is therefore necessary.

**Electronic supplementary material:**

The online version of this article (doi:10.1186/s12875-014-0182-9) contains supplementary material, which is available to authorized users.

## Background

The clinical exercise test has been validated for diagnosing ischaemic heart disease in primary care [1,2]. The sensitivity and specificity of an exercise test for detecting coronary artery disease, using coronary angiography as a reference, are approximately 70% and 80%, respectively. There is, however, a wide variability in accuracy [3]. The utility of exercise tests depends not only on the accuracy of the test per se, but also on the ability of the test to support treatment decisions. The prognostic reliability of a negative exercise test was reported in a two-year follow-up conducted in Finnish primary care; 2% of patients aged less than 60 years and 3% of patients aged 60 years and older were diagnosed with coronary disease at the end of follow-up [4,5]. As a diagnostic instrument for coronary disease, the usefulness of exercise testing depends on patient characteristics such as age, gender, and type of chest pain; best diagnostic yield is achieved when the pre-test probability of coronary disease is intermediate (15-65%) [6,7]. We reported previously that primary care patients with negative exercise tests only had a 2% risk of cardiovascular events, compared to a 52.7% risk in patients with positive tests within six months of follow-up [8]. In another primary care-based study of chest pain patients, ischaemic heart disease was confirmed or excluded in 77% of patients after exercise testing [9]. Such information may be helpful in the clinical decision process.

Medical decisions are usually based on estimates of pre-test probability, which may be affected by irrelevant conditions, resulting in biased decisions that may be problematic for patients and providers of health care. Gender bias is defined as “any non intentional, but systematic, discrimination of women or men” [10]. Gender bias in the diagnosis and treatment of cardiovascular disease has been reported by many research groups [11-16], but others failed to identify such bias [17-20] or found equivocal results [21]. For example, women with angina pectoris were less likely to receive combined drug therapy (aspirin, beta-blockers, and statins) and less likely to access exercise electrocardiography (ECG) and revascularisation in a cross-sectional study from British primary care in 2001 [13]. In an Irish population-based cohort study conducted in 2000–2001, women were enrolled in secondary prevention programmes less frequently than men [11]. In a meta-analysis of cardiac rehabilitation programmes (19 studies published from 2000 to 2011; 241 613 enrolled patients), women were less likely to be referred to cardiac rehabilitation than men [12]. According to a prospective study conducted in 74 German primary care clinics in 2005–2006, male patients received more exercise tests and hospital admissions, but after adjustment for chest pain character there was no significant difference [21]. In a study based on the Swedish Discharge Register of patients treated for coronary disease during 1991–2000, men were 1.5 times more likely to undergo revascularisation than women, and compared to other men, men of lower employment grades had less access to coronary artery bypass grafting [15]. In a British study of 1522 patients referred to a chest pain clinic for exercise testing or angiography in 1997–2000, there was no under-investigation of women, with the exception of a lower predictive value of exercise tests in women than men [20]. A study from the Spanish primary care setting in 2006 found that screening of cardiovascular risk factors was equal among men and women, but men received more prescriptions for secondary preventive drugs [14]. In a Norwegian cohort of 931 women and 2174 men treated for myocardial infarction in 2006–2007, women with ST-elevation infarctions received similar treatment as men, but women with non-ST-elevation infarctions were less likely to undergo angiography or to have a percutaneous coronary intervention [16].

Socioeconomic status can be defined in many ways, such as by educational level, occupation, and income [22]. Employment grade and educational level may be used to cover socioeconomic status from different aspects, since medical decisions may be affected by the patient’s socioeconomic background [23]. However, a report from the Whitehall II cohort uncovered no evidence that low socioeconomic status was associated with less use of cardiac diagnostic procedures or drugs, given the inverse gradient in coronary morbidity between individuals of low and high socioeconomic status [24]. Obviously, all phases of cardiovascular research in the primary care setting should consider the influences of gender and socioeconomic status.

Our primary objective in this study was to assess the utility of exercise tests in selecting primary care patients for referral to further cardiologic evaluation. Our other objectives were to identify whether referral decisions were biased by gender or socioeconomic status and to describe referrals and cardiovascular events in patients with positive exercise tests.

## Methods

### Design

We designed a prospective observational study of primary care patients referred to clinical exercise testing due to a suspicion of ischaemic heart disease. We previously described the details of this study cohort [8].

### Setting

We recruited patients from 28 primary care clinics that served an adult population (age ≥ 20 years), of approximately 99 000 inhabitants in 2012, in the County of Jämtland in the northern part of Sweden. Forty seven percent of the study population lived in the central municipality of Östersund, with the remainder in various rural municipalities; data provided from Statistics Sweden. Exercise tests were performed at the Department of Clinical Physiology, Östersund Hospital. After exercise testing, general practitioners (GPs) had the option to refer patients to the Department of Cardiology, Östersund Hospital. There were no other external providers of cardiologic services taking referrals from GPs during the study period. All engaged GPs were employed by the county or had contracts for primary care services with the county council. The Department of Cardiology, Östersund Hospital, referred patients for revascularisation to the University Hospital in Umeå at the time of the study.

### Recruitment and follow-up

We invited potentially eligible patients referred to exercise testing from GPs from February 2010 until the end of February 2012 to participate in the current investigation. At the time of the study there were no local guidelines to support GPs in the use of exercise tests or for referral to a cardiologist after testing. Enrolled study patients were referred due to suspected ischaemic heart disease at the discretion of the GPs who evaluated patients that accessed primary care. Of the 1191 potentially eligible patients, 265 declined to provide consent, eight were unable to carry out exercise testing, and 53 were referred for reasons other than ischaemic heart disease. The study group thus consisted of 865 patients, 438 men and 427 women. It was possible to follow all participants through the electronic medical record system within six months of exercise testing. Registered events were: patients referred within six months to cardiologic evaluation, patients with hospitalisation for myocardial infarction, or hospitalisation for unstable angina and cases of cardiovascular death. Revascularisations (coronary artery bypass grafting or percutaneous coronary intervention) were recorded within 250 days from exercise testing, in some cases due to delays between referral and revascularisation. Myocardial infarctions were diagnosed in accordance with the universal definition [25].

Follow-up of study patients was performed by GN and one assistant via the electronic medical record system. Records were provided from patients migrating to other counties during the study period. There was no blinding to the outcome of exercise tests or to other characteristics, since the records were scrutinised in complete form. Our aim was to reflect normal care in which the exercise test results are signed and approved by a GP at the referring unit.

### Measurements and classifications

Along with the notice for exercise testing, we mailed a questionnaire to be completed by the patients before the exercise test. The questionnaire addressed previous medical history, medication, smoking habits, chest-pain symptoms, educational level, and employment status. The three questions on chest pain were “Do you ever have chest pain or discomfort in the chest?”, “Do you have chest pain walking at an ordinary pace on the level?”, and “Do you have chest pain walking uphill or in a hurry?” The three chest pain questions were previously published as a part of the “Rose angina questionnaire” [26,27]. In our study, positive answers to the second or third chest-pain questions were summarised as “exertional chest pain”. Questions on educational level and other baseline characteristics were provided with fixed alternatives.

We used the Swedish socioeconomic classification (SEI) [28] to assign the socioeconomic status of study participants. SEI classification is based on the patient’s main occupational background and level of qualification. Within the SEI classification, it is also possible to classify unemployed and self-employed individuals; retirees are classified by their previous main occupation. We used an aggregated version of the SEI that contained main categories of manual workers, non-manual employees, and self-employed/employers. Employed patients consisted of manual workers and non-manual employees. The aggregated version of the SEI classification is presented in Additional file 1. Retired participants were classified within the SEI system and not in a separate category; this classification also applied to participants on sick leave and to participants who were part-time retired.

Before the exercise test, we recorded a resting ECG and supine systolic and diastolic blood pressure in all study patients. Resting ECGs were classified by the physician responsible for the test procedure, in accordance with Minnesota Code guidelines [8,29]. Exercise tests were performed as a bicycle test, in accordance with national guidelines; a complete description of the exercise test procedure and classification has been published elsewhere [8,30-32].

### Exercise test classification

Positive exercise tests were associated with a depression of the ST segment >0.1 mV, horizontal or down-sloping, and chest pain indicating angina during the test. Inconclusive tests were associated with either chest pain or ST-segment depression during the test. Patients with negative tests experienced neither chest pain nor ST-segment depression. ECG reactions impossible to assess due to left bundle branch block, pacemaker, or digitalis medication were classified as non-assessable.

### Statistical methods

Patient characteristics are reported as proportions and means. As appropriate, between group comparisons were evaluated via Student’s *t-*test, the chi-squared test, or Fisher’s exact test (two-sided). We used univariate logistic regression to identify characteristics that were associated with referral to cardiologic examination. In the prediction model, baseline characteristics were entered into a multivariable logistic model and reduced stepwise by exclusion of the least-significant variable until only significant variables remained. In the final model, independently significant variables were adjusted for age and gender. To detect potential interactions between gender and socioeconomic status, we constructed adjusted models of male and female patients separately. The level of significance was p < 0.05. Statistical analyses were carried out with IBM SPSS version 22.

### Ethical approval

Ethical approval was obtained from the Regional Ethical Review Board at Umeå University. All participants in the study provided written informed consent.

## Results

### Descriptive data

The mean age of the study population (427 women and 438 men) was 63.5 years; women were approximately two years older than men. Primary education was the highest educational level in 46.3% of women and 48.5% of men. Manual work was the predominant socioeconomic class (women 46.9%, men 49.9%). Fewer women were self-employed (8.3%) than men (18.8%; p < 0.001). Self-employed study participants most often ran single-person companies (63.5%), and only 8% of self-employed participants hired five persons or more. Agriculture and forestry (25%), trading (21%), service (16%), and transport (11%) were the most common types of enterprise. Previous cardiovascular events were less common in women (11.2%) than in men (21.3%). Characteristics of self-employed men and women are provided in Additional file 2. We previously reported the medical characteristics of the complete study cohort [8].

## Main results

**Figure 1:**
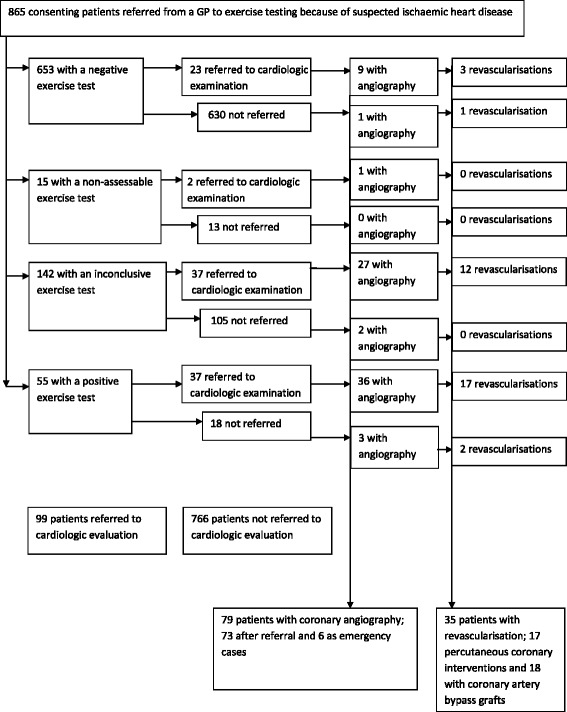
**Study profile of participant recruitment and events within 250 days of clinical exercise testing.**

Of 865 study patients examined with clinical exercise testing upon referral from GPs, all completed follow-up. By six months, 99 patients were referred from primary care to evaluation at a heart clinic. Seventy-nine patients underwent coronary angiography, 73 by referral from a GP to a cardiologist and six as emergency cases (Figure 1). In 63.3% of all patients with coronary angiography, coronary disease was confirmed. Thirty-five patients, 25 men and 10 women, had a revascularisation within six months (p = 0.012). Patients with positive (n = 55), inconclusive (n = 142), negative (n = 653), or non-assessable (n = 15) exercise tests were referred to further cardiologic evaluation in 67.3%, 26.1%, 3.5%, and 13.3% of cases, respectively. Fewer women (9.4%) than men (13.5%) were referred to cardiologic evaluation, but this difference was not significant (Table 1).

**Table 1:** **Proportions of patients referred to cardiologists, categorised by exercise-test result, educational level, and socioeconomic classification**

**Characteristic**	**Number of patients referred/number of patients with characteristic (%)**			**P value for difference**
	**Women**	**Men**	**Total**	**0.058**
	**40/427 (9.4%)**	**59/438 (13.5%)**	**99/865 (11.4%)**	
**Exercise test result**				
Positive test	11/19 (57.9%)	26/36 (72.2%)	37/55 (67.3%)	0.282
Inconclusive test	17/72 (23.6%)	20/70 (28.6%)	37/142 (26.1%)	0.501
Negative test	11/328 (3.4%)	12/325 (3.7%)	23/653 (3.5%)	0.814
Non-assessable test	1/8 (12.5%)	1/7 (14.3%)	2/15 (13.3%)	1.000
**Educational level**				
Primary education	18/176 (10.2%)	26/192 (13.5%)	44/368 (12.0%)	0.328
Secondary education	9 /118 (7.6%)	17/152 (11.2%)	26/270 (9.6%)	0.326
University or college degree	8/86 (9.3%)	6/52 (11.5%)	14/138 (10.1%)	0.673
Missing data	5/47 (10.6%)	10/42 (23.8%)	15/89 (16.9%)	0.098
**Socioeconomic classification**				
Manual workers	21/180 (11.7%)	26/204 (12.7%)	47/384 (12.2%)	0.748
Non-manual employees	10/172 (5.8%)	19/128 (14.8%)	29/300 (9.7%)	0.009
Self-employed	6/32 (18.8%)	10/77 (13.0%)	16/109 (14.7%)	0.553
Missing data	3/43 (7.0%)	4/29 (13.8%)	7/72 (9.7%)	0.429

Ninety-nine primary care patients referred to a cardiologist out of 865 patients examined with clinical exercise testing due to suspected coronary disease. Referrals were recorded within six months from exercise testing.

Overall, neither employment status nor socioeconomic level was discriminatory for referral to cardiologic evaluation, but for referrals we detected a positive interaction between gender and employment status. Among non-manual employees, women were referred to cardiologic examination less frequently (5.8%) than men (14.8%; p = 0.009; Table 1); the difference remained significant after adjustment for exercise test result, exertional chest pain, and age (odds ratio (OR) 0.40, 95% confidence interval (CI) 0.16-1.00; p = 0.049).

Self-employment predicted referral in women (OR 3.62, 95% CI 1.19-10.99, adjusted for age, exertional chest pain, and exercise test result), with employed females as reference. Compared to employed women, employed men had a higher OR for referral, but not significantly (OR 1.73, 95% CI 0.99-3.01). Employed and self-employed men did not have significantly different ORs for referral (Table 2). Self-employed women had less often been subject to a revascularisation (0%) compared to self-employed men (13.2%; p = 0.032) (Additional file 2). In other characteristics there were no significant differences between self-employed men and women.

**Table 2:** **Adjusted ORs (95% CIs) for referral to cardiologic evaluation, by gender and employment**

**Gender**	**Employment**	
	**Employed**	**Self-employed**
**Female**	1.00 (n = 352)	3.62 (1.19-10.99) (n = 32)
**Male**	1.73 (0.99-3.01) (n = 332)	1.87 (0.76-4.61) (n = 77)

Referrals took place within six months from exercise testing. Employed females served as reference. ORs were adjusted for age, exertional chest pain, and positive/inconclusive exercise test result versus negative test.

Referred women more often had a previous revascularisation or myocardial infarction, and they had slightly higher systolic blood pressure, compared to non referred women. Among men, referred patients were older, had a lower body mass index, and were more frequently on medication for hypertension. Referred men reported chest-pain symptoms more frequently than non referred men. Men and women referred to cardiologic examination had exertional chest pain, angina according to their own assessment, and a pathologic ST-T segment on resting ECG more frequently than non-referrals. A positive exercise test was the most powerful predictor for referral to a cardiologist in women (OR 17.97, 95% CI 6.71-48.16) and in men (OR 29.07, 95% CI 12.91-65.46), but inconclusive tests also predicted referral . The unadjusted ORs and p values for referral of women and men, according to patient characteristics, are provided as additional data (Additional file 3).

In a multivariable model of patient characteristics associated with referral to cardiologic evaluation, a positive/inconclusive exercise test and exertional chest pain were associated with referral, adjusted for age and gender (Table 3). In female patients, a positive/inconclusive exercise test, exertional chest pain, previous revascularisation, and self-employment were associated with referral, adjusted for age (Table 4); in male patients, a positive/inconclusive exercise test, exertional chest pain, and a pathologic ST-T segment predicted referral (Table 4).

**Table 3:** **Adjusted ORs for referral to cardiologists according to patient characteristics (n = 865)**

**Patient characteristic**	**OR (95% CI)**	**P**
Positive/inconclusive exercise test	12.43 (7.49-20.64)	<0.001
Exertional chest pain	2.71 (1.57-4.68)	<0.001
Age	1.00 (0.98-1.03)	0.717
Female gender	0.66 (0.41-1.08)	0.096

Characteristics remaining significant in a multivariable analysis adjusted for age and gender. Follow-up within six months of exercise testing.

**Table 4:** **Adjusted ORs for referral to cardiologist according to patient characteristics in women and men**

**Patient characteristic**	**Women (n = 427)**		**Men (n = 438)**	
	**OR (95% CI)**	**P**	**OR (95% CI)**	**P**
Positive/inconclusive exercise test	11.07 (5.11-23.95)	<0.001	13.60 (6.77-27.33)	<0.001
Exertional chest pain	2.40 (1.03-5.63)	0.043	3.22 (1.52-6.85)	0.002
Pathologic ST-T segment on resting ECG	-	NS	2.42 (1.07-5.49)	0.034
Previous revascularisation	5.15 (1.33-19.97)	0.018	-	NS
Self-employed	3.92 (1.29-11.92)	0.016	-	NS
Age	0.99 (0.96-1.03)	0.589	1.01 (0.98-1.04)	0.655

Characteristics remaining significant in a multivariable analysis adjusted for age. Follow-up within six months of exercise testing. NS = not significant.

Among self-employed patients, the number of employees was not associated with referral (p = 0.478). Among employed participants, the OR for referral of women, compared to men, was close to significance (OR 0.58, 95% CI 0.33-1.01; p = 0.053) after adjustment for exercise test result, exertional chest pain, and age (employed participants are patients with SEI classification 11–57 for manual workers and non-manual employees, Additional file 1). Referral did not differ by employment grade (SEI classification 11–57) for either sex.

In patients with positive exercise tests, the probability of referral to cardiologic evaluation diminished with increasing age, from 100% for ages 40–49 years to 40% for ages 80 years and older (p value for trend = 0.024; Table 5). This age gradient remained significant after adjustment for co-morbidity (previous revascularisation, myocardial infarction, transitory ischaemic attack, stroke, or exertional chest pain; OR 0.48, 95% CI 0.23-0.97; p = 0.042).

**Table 5:** **Patients with positive exercise tests, by age and referral to cardiologic evaluation within six months**

**Age in years**	**Exercise test positive**	
	**Referred to cardiologist (%)**	**Not referred to cardiologist (%)**
**40-49**	4 (100%)	0 (0%)
**50-59**	5 (83.3%)	1 (16.7%)
**60-69**	14 (73.7%)	5 (26.3%)
**70-79**	12 (57.1%)	9 (42.9%)
**80-89**	2 (40%)	3 (60%)
**All ages**	37 (67.3%)	18 (32.7%)

Fifty-five primary care patients with positive exercise tests, of 865 patients examined; two-sided exact test for trend, p = 0.024.

Among 18/55 patients with positive exercise tests and no further referral, four cardiovascular events (22.2%) occurred during follow-up (Table 6). Two patients (11%) had emergency revascularisations and six patients (33%) were judged not to require invasive treatment (stable angina with few symptoms on medication) by their GPs. In 10 cases (56%), the records did not provide any data about medical decisions based on the exercise test result.

**Table 6:** **Patients with positive exercise tests by age, gender, cardiovascular events and referral to cardiologic evaluation**

**Age**	**Exercise test positive**		
	**Referred to cardiologist (%); mean (SD) n = 37**	**Not referred to cardiologist (%); mean (SD) n = 18**	P*
**Age in years, mean (SD)**	65.7 (10.5)	70.9 (7.0)	0.058
**Female gender**	11 (29.7%)	8 (44.4%)	0.368
**Any cardiovascular event**	21 (56.8%)	4 (22.2%)	0.022
Revascularisation	17 (45.9%)	2 (11.1%)	0.015
Myocardial infarction, hospitalisation for	1 (2.7%)	0 (0.0%)	1.000
Unstable angina, hospitalisation for	9 (24.3%)	1 (5.6%)	0.140
Cardiovascular death	0 (0.0%)	1 (5.6%)	0.327

Fifty-five primary care patients with positive exercise tests, out of 865 patients examined. SD = standard deviation. *Fisher’s exact test (two-sided) or Student’s *t*-test, as applicable.

## Discussion

### Key results

In this study of patients referred to exercise testing for suspected coronary disease, the exercise test result (positive/inconclusive tests vs. negative tests) was strongly associated with referral to cardiologic evaluation (Table 3). Overall, there were no significant differences in referral rates related to gender, socioeconomic status, or age. However, we detected interactions between sex and socioeconomic status in the referral rates for cardiologic evaluation after exercise testing. Self-employed women were more likely to be referred to cardiologic evaluation than other women; among men, there was no such interaction (Table 2). Among non-manual employees, female patients were referred to cardiologic evaluation less frequently than male patients, even after adjustment for confounding factors.

Among female patients, exercise test results, exertional chest pain, previous revascularisations, and self-employment were all independently associated with referral. Among male patients, exercise test results, exertional chest pain, and ST-T segment pathology on resting ECG were associated with referral (Table 4). In patients with positive exercise tests, the referral rate decreased with age (Table 5). We could not find any consideration of the result in the records for half of the exercise test-positive patients who were not referred to cardiologic evaluation.

### Strengths and limitations

We recruited patients from a population with access to tax-funded health care with a small share of self-payment. This setting captured the normal level of care for patients consulting for suspected angina. All enrolled patients were followed up, and exercise tests were performed in a single laboratory. All patients considered to be in need of secondary cardiologic evaluation were referred to the Cardiology Unit at Östersund Hospital; there were no private providers of cardiologic services within the study area. We did not use an external expert panel for reference diagnosis of ischaemic heart disease, since we wished the study results to reflect the standard of normal care.

Compared to the complexity of coronary disease, any classification system based on exercise tests is a simplification. Classification into three categories yields a more complex result than classification into positive and negative tests. However, in the regression analysis of referrals, we implemented a bivariate approach because both positive and inconclusive test results need to be considered for patient management.

There was no blinding of outcome data, relative to patient characteristics, since all medical records were scrutinised in complete form. Patient characteristics and outcome data were determined independently from each other to avoid recall or classification bias within the observational study design.

Medication was registered from the pre-test questionnaire. The use of medication lists in records could have been another possible study strategy, but medication lists are not always up to date, and patient compliance may be unreliable.

We used employment status and educational level as measurements of socioeconomic status. We chose not to use other measures of socioeconomic status, such as income, liquid assets, and housing conditions [33], since economic and housing measurements could be regarded as sensitive information by the patients and affect their willingness to participate. Since retirement was not recorded as a separate patient characteristic, we could not analyse the influence of retirement on referral to cardiologic evaluation, separated from age, or possible differences in retirement age in different socioeconomic groups. The educational level in the study population was low, and self-employed patients predominantly ran smaller companies. The generalisability of our findings must be considered with respect to socioeconomic conditions, demography, and availability of health care.

The reasons for not referring patients with positive exercise tests were frequently not reflected by the medical records. Other study designs, such as focus-group interviews with a qualitative approach, could have been helpful in elucidating these questions about the referral of patients from GPs to cardiologists.

We did not use a panel group for reference diagnosis in patients that did not undergo further cardiology work-up. Some cases of significant coronary disease probably remained undetected in patients who were not further examined after exercise testing. In an observational study such as this one, it is not possible to avoid that type of limitation. Some of our findings are close to the level of significance or are based on small numbers. Conclusions based on such results must be supported by other studies, preferably with larger samples, to allow for more robust conclusions. It is also possible that the number of observations in our cohort was too small to detect a referral bias.

### Interpretation of findings

Several explanations may underlie the observed interaction between gender and socioeconomic status for referral to cardiologic evaluation. For example, self-employed women may be treated differently from self-employed men because self-employment is perhaps closer to the prevailing masculine norm. Other explanations are possible, such as differences in information and promotion of actions for health care in these patient groups.

Among employed patients, the difference in ORs for referral between men and women was borderline significant (OR 1.73, 95% CI 0.99-3.01). Thus, our findings indicate that employed men may have better access to advanced cardiologic care than employed women. Differences in access to cardiologic care by referral from GPs were previously reported by others [13].

Our results highlight the importance of using various measures of socioeconomic status, since different socioeconomic measures are not assumed to be interchangeable [33]. Different measures may be of different importance among various social groups [33], as well as within groups categorised by age [34] or by sex [35,36]. Socioeconomic status can be understood as a multidimensional construct of various factors (economic resources, education, occupational level, deprivation of neighbourhood resources) that operates through a variety of pathways [33,37-40].

Previous revascularisation was associated with referral among women, but not men. The reason for this difference is not clear. From an epidemiologic perspective, a female patient may be less likely to have an obstructive coronary disease than a male patient with similar symptoms and age [6,7]; from a another perspective, GPs may be influenced by gender stereotypes that affect their medical decisions [41]. GPs may also find it more difficult to evaluate symptoms and physiologic test results in women, and therefore refer women more often when there is clear evidence of coronary disease in the patient’s history.

The reasons patients with a positive exercise test were not referred for cardiology evaluation remain unclear; in 56% of such cases, the records did not provide any data reflecting the test result or actions taken. One possible contributing cause is continuity problems in primary care, with GPs working on short-term contracts. A more thorough exploration of this issue is beyond the scope of the present investigation.

### Relevance of findings

We identified complex interactions between socioeconomic status and gender in terms of referral rate. Among non-manual workers, women were less frequently referred to cardiologic examination than men; self-employed women were referred more often than other women. Age was an important predictor of referral when the exercise test result was positive. These findings raise several concerns. We need to be more aware of possible biases involving age, gender, and socioeconomic status that may influence important medical decisions in our daily work as GPs. Here, exercise test results were important for decisions to refer patients to cardiologic evaluation. This picture, derived from a Swedish primary care setting in 2010–2012, is likely to change when new guidelines are implemented [7] and the availability of new and more sensitive imaging modalities increases [42,43]. The interaction between gender and employment status demonstrated here should be confirmed in other cohorts.

## Conclusions

Exercise tests are important for selecting patients for referral to cardiologic care. Interactions between gender and socioeconomic status affected referral rates. In patients with positive exercise tests, referral rates decreased with age. Patients with a positive stress test are at high risk for cardiovascular events, and reasons for non-referral should be appropriately documented. An increased awareness of possible biases regarding age, gender, and socioeconomic status, which may influence medical decisions, is necessary.

## Additional files

Additional file 1:
**Swedish socioeconomic classification (SEI).** Published in Reports on Statistical Co-ordination 1982:4, Statistics Sweden. Additional file 2:
**Characteristics of self-employed men and women.**
Additional file 3:
**Crude ORs for referral to cardiologists within six months of clinical exercise testing.**
