# Supplementary material for: Use of exercise tests in primary care: importance for referral decisions and possible bias in the decision process; a prospective observational study
Source: BMC Fam Pract. 2014 Nov 30;15:182. doi: 10.1186/s12875-014-0182-9 (PMC4276015; doi:10.1186/s12875-014-0182-9)
Supplement: Additional file 1: — Swedish socioeconomic classification (SEI). Published in Reports on Statistical Co-ordination 1982:4, Statistics Sweden. [file 12875_2014_182_MOESM1_ESM.pdf]

Additional file 1

Swedish socioeconomic classification (SEI)*

Manual workers: Occupations normally organised by The Swedish Trade Union Confederation.

	Delineations
11 Unskilled employees in goods production	Less than 2 years of post-comprehensive school education
12 Unskilled employees in service production	Less than 2 years of post-comprehensive school education
21 Skilled employees in goods production	2 years or more of post-comprehensive school education
22 Skilled employees in service production	2 years or more of post-comprehensive school education

Non-manual employees: Occupations normally organised by The Swedish Confederation of Professional Employees or The Swedish Confederation of Professional Associations.

33 Assistant non-manual employees, lower level	Less than 2 years of post-comprehensive school education
36 Assistant non-manual employees, higher level	2 but not 3 years of post-comprehensive school education
46 Intermediate non-manual employees	3 but not 6 years of post-comprehensive school education
56 Professionals and other higher non-manual employees	At least 6 years of post-comprehensive school education
57 Upper-level executives	Upper-level executives in private enterprises or organisations with at least 100 employees or in public service

Employers	Delineations
60 Self-employed professionals	At least 6 years of post-comprehensive school education
79 Self-employed other than professionals and farmers	-
89 Farmers	

* Published in Reports on Statistical Co-ordination 1982:4, Statistics Sweden [24].
