# Supplementary material for: Use of exercise tests in primary care: importance for referral decisions and possible bias in the decision process; a prospective observational study
Source: BMC Fam Pract. 2014 Nov 30;15:182. doi: 10.1186/s12875-014-0182-9 (PMC4276015; doi:10.1186/s12875-014-0182-9)
Supplement: Additional file 2: — Characteristics of self-employed men and women. [file 12875_2014_182_MOESM2_ESM.pdf]

Additional file 2 Characteristics of self-employed men and women

Characteristic	Women n=32	Men n=77	P
**Age in years, mean (SD)**	65.0 (8.6)	61.6 (11.7)	0.139
**Male GP**	17 (53.1%)	55 (71.4%)	0.078
**Body mass index in kg/m^2, mean (SD)**	26.8 (4.8)	27.1 (3.4)	0.708
**Systolic blood pressure in mmHg, mean (SD)**	149 (21)	149 (21)	0.985
**Diastolic blood pressure in mmHg, mean (SD)**	84 (12)	84 (9)	0.869
**Educational level**			
Primary education	13 (40.6%)	38 (51.4%)	0.398
Secondary education	15 (46.9%)	28 (37.8%)	0.398
University or college degree	4 (12.5%)	8 (10.8%)	0.751
**Single-person enterprise**	14 (77.8%)	33 (58.9%)	0.172
**Current smoker**	1 (3.1%)	4 (5.2%)	1.000
**Past medical history**			
Myocardial infarction	2 (6.5%)	11 (14.3%)	0.340
Revascularisation	0 (0%)	10 (13.2%)	0.032
Stroke or transient ischaemic attack	1 (3.1%)	5(6.6%)	0.667
**Present conditions**			
Hypertension, medication for	17 (54.8%)	42 (54.5%)	1.000
Diabetes mellitus, treatment for	4 (12.9%)	10 (13.0%)	1.000
Dyslipidaemia, medication for	7 (22.6%)	22 (28.9%)	0.633
Congestive heart failure, medication for	3 (10.0%)	12 (16.0%)	0.547
Claudication	1 (3.3%)	6 (8.0%)	0.670
**Chest pain symptoms**			
Ever have chest pain or discomfort in the chest	24 (80.0%)	47 (61.8%)	0.108
Exertional chest pain	18 (56.3%)	38 (51.4%)	0.677
**Angina diagnosis according to patient**	6 (20.7%)	18 (25.0%)	0.798
**Resting ECG**			
Normal resting ECG	25 (78.1%)	58 (75.3%)	0.810
Atrial fibrillation	0 (0%)	6 (7.8%)	0.177
Pathologic Q wave	0 (0%)	7 (9.1%)	0.103
Pathologic ST-T segment	2 (6.3%)	4 (5.2%)	1.000
**Exercise test result**			
Positive test	1 (3.1%)	4 (5.2%)	1.000
Inconclusive test	5 (15.6%)	10 (13.0%)	1.000
Negative test	25 (78.1%)	62 (80.5%)	0.797
Non-assessable test	1 (3.1%)	1 (1.3%)	0.503
Positive/inconclusive test	6 (18.8%)	14 (18.2%)	1.000

P values were calculated by Fisher's exact test (two-sided) or Student's *t*-test, as applicable.

SD, standard deviation.
