# Supplementary material for: Use of exercise tests in primary care: importance for referral decisions and possible bias in the decision process; a prospective observational study
Source: BMC Fam Pract. 2014 Nov 30;15:182. doi: 10.1186/s12875-014-0182-9 (PMC4276015; doi:10.1186/s12875-014-0182-9)
Supplement: Additional file 3: — Crude ORs for referral to cardiologists within six months of clinical exercise testing. [file 12875_2014_182_MOESM3_ESM.pdf]

Additional file 3 Crude ORs for referral to cardiologists within six months of clinical exercise testing

**Patient characteristic**	**Women (n=427)**		**Men (n=438)**	
	**OR (95% CI)**	**P**	**OR (95% CI)**	**P**
**Age in years**	1.01 (0.98-1.04)	0.490	1.03 (1.01-1.06)	0.013
**Referral by a male GP**	1.33 (0.67-2.66)	0.421	1.12 (0.60-2.11)	0.715
**Educational level**				
Primary education	1.25 (0.63-2.51)	0.525	1.23 (0.68-2.24)	0.494
Secondary education	0.75 (0.34-1.65)	0.475	0.83 (0.45-1.56)	0.571
University or college degree	1.01 (0.44-2.32)	0.973	0.91 (0.37-2.27)	0.844
**Socioeconomic classification**				
Manual workers	1.55 (0.78-3.08)	0.208	0.89 (0.50-1.57)	0.678
Non-manual employees	0.42 (0.20-0.90)	0.026	1.19 (0.65-2.16)	0.577
Self-employed	2.39 (0.91-6.25)	0.076	0.95 (0.46-1.99)	0.895
**Current smoker**	0.72 (0.21-2.44)	0.594	0.84 (0.29-2.48)	0.754
**Body mass index in kg/m^2**	1.03 (0.97-1.10)	0.355	0.93 (0.86-1.00)	0.044
**Systolic blood pressure in mmHg**	1.02 (1.00-1.04)	0.013	1.01 (0.99-1.02)	0.404
**Diastolic blood pressure in mmHg**	1.01 (0.97-1.04)	0.715	0.98 (0.96-1.01)	0.288
**Past medical history**				
Myocardial infarction	3.51 (1.21-10.24)	0.021	0.90 (0.39-2.08)	0.797
Revascularisation	7.39 (2.48-22.01)	<0.001	1.37 (0.65-2.87)	0.410
Stroke or transient ischaemic attack	2.01 (0.65-6.21)	0.224	1.95 (0.75-5.04)	0.171
**Present conditions**				
Hypertension, medication for	1.87 (0.95-3.69)	0.072	1.86 (1.03-3.57)	0.039
Diabetes mellitus, treatment for	1.42 (0.52-3.86)	0.491	0.90 (0.39-2.08)	0.798
Dyslipidaemia, medication for	1.55 (0.76-3.20)	0.232	1.27 (0.72-2.25)	0.418
Congestive heart failure, medication for	1.47 (0.58-3.72)	0.413	1.22 (0.56-2.64)	0.619
Claudication	0.91 (0.21-4.09)	0.913	1.24 (0.41-3.75)	0.705
**Chest-pain symptoms**				
Ever have chest pain	1.07 (0.49-2.33)	0.870	1.98 (1.05-3.74)	0.035
Exertional chest pain	3.56 (1.65-7.68)	0.001	5.03 (2.63-9.63)	<0.001
**Angina according to patient's own assessment**	3.03 (1.53-6.00)	0.001	3.17 (1.76-5.70)	<0.001
**Resting ECG**				
Normal resting ECG	0.52 (0.26-1.06)	0.073	0.66 (0.37-1.06)	0.146
Atrial fibrillation	0.97 (0.12-7.75)	0.975	1.13 (0.38-3.38)	0.833
Pathologic Q wave	0.87 (0.20-3.86)	0.858	0.81 (0.31-2.14)	0.667
Pathologic ST-T segment	2.67 (1.18-6.02)	0.018	2.60 (1.34-5.05)	0.005
**Exercise test result**				
Positive test	17.97 (6.71-48.16)	<0.001	29.07 (12.91-65.46)	<0.001
Inconclusive test	4.46 (2.24-8.88)	<0.001	3.37 (1.82-6.25)	<0.001
Positive/inconclusive test	12.00 (5.79-24.85)	<0.001	18.81 (9.58-36.93)	<0.001
Negative test	0.08 (0.04-0.18)	<0.001	0.05 (0.03-0.11)	<0.001

Ninety-nine patients (40 women and 59 men) referred to cardiologists out of 865 patients examined with clinical exercise testing due to suspected coronary disease.
